# Off-label use of commercially available topical sirolimus for keratosis pilaris rubra faciei

**DOI:** 10.1016/j.jdcr.2024.10.031

**Published:** 2024-11-23

**Authors:** Katherine P. Snow, Samantha K. Ong, Elaine Siegfried

**Affiliations:** a Department of Dermatology, Saint Louis University School of Medicine, St. Louis, Missouri; b Department of Dermatology, SSM Health Saint Louis University Hospital, St. Louis, Missouri; c Division of Dermatology, Department of Pediatrics, SSM Health Cardinal Glennon Children’s Hospital, St. Louis, Missouri

**Keywords:** dermatology, erythromelanosis follicularis faciei et colli, keratosis pilaris, keratosis pilaris rubra faciei, novel, pharmacology, sirolimus, topical, treatment

## Introduction

Keratosis pilaris rubra faciei (KPRF), considered a subtype of keratosis pilaris, has a characteristic appearance featuring follicular-based fine hyperkeratotic papules and prominent background erythema in a patterned distribution, with associated rough texture.^1^^,^^2^ Patients with KPRF are often distressed by the appearance and tactile change as well as frequent flushing, burning, and stinging.^3^^,^^4^

Oral sirolimus, a rat sarcoma pathway-associated mammalian target of rapamycin kinase inhibitor, was initially United States Food and Drug Administration-approved in 1999 for organ transplant rejection prophylaxis. It has also been used off-label for a variety of skin conditions that feature vascular prominence, including vascular malformations, cutaneous graft-versus-host disease, and tuberous sclerosis-associated facial angiofibromas. Subsequent use of extemporaneously compounded topical 0.003% to 3% sirolimus in a variety of vehicles was reported as early as 2011 to treat facial angiofibromas.^5^ Unclear stability and bioavailability of compounded formulations prompted development and ultimate United States Food and Drug Administration-approval of a 0.2% sirolimus in an optimized gel vehicle, for tuberous sclerosis-associated facial angiofibromas.

A 2022 case report documented dramatically improved facial erythema in a 15-year-old male using twice-daily extemporaneously compounded topical 1% sirolimus cream.^4^ This case report documents similar success using commercially available 0.2% gel (Hyftor), supporting the value of a clinical trial.

## Case

**Fig 1:**
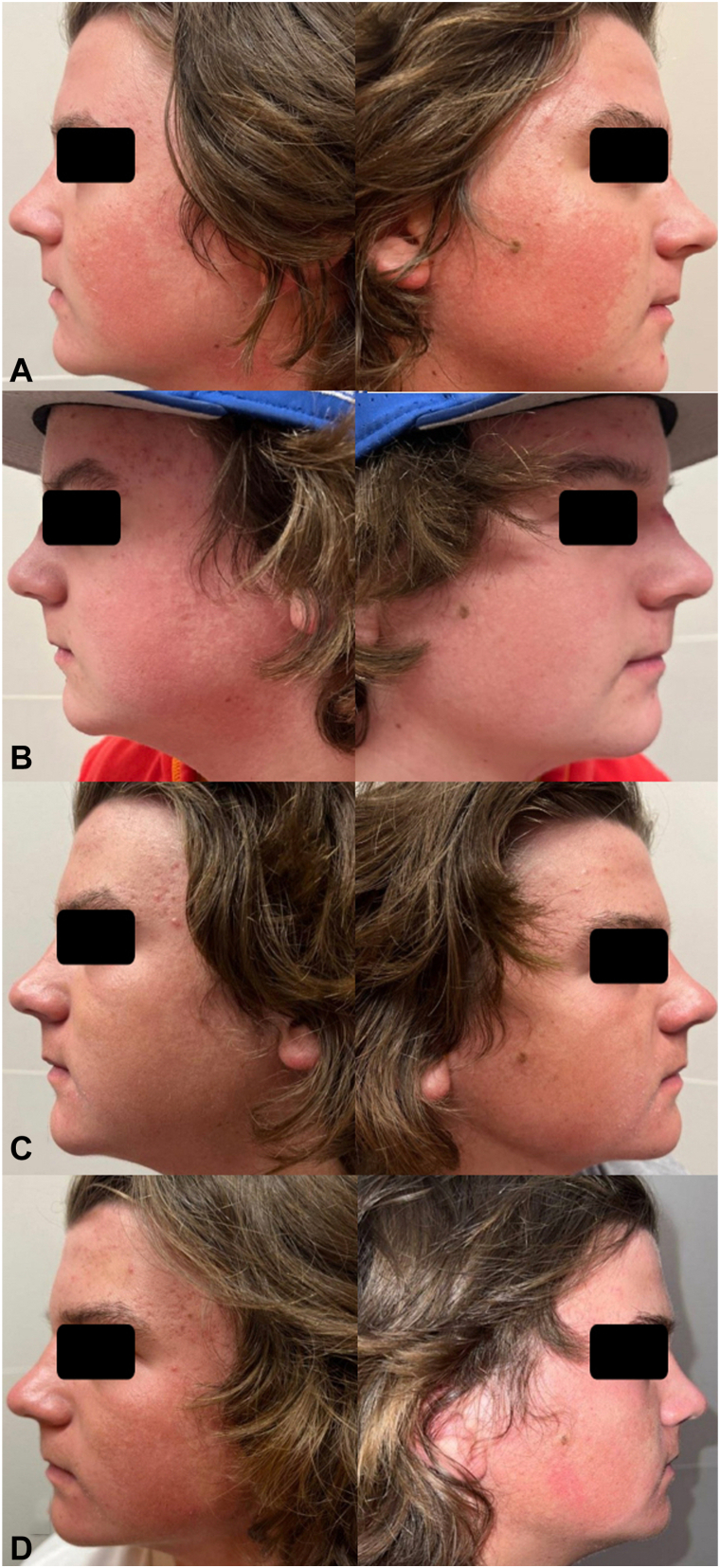
**A,** Baseline. **B,** Improvement following a 3-month course of twice daily application of topical sirolimus 0.2% gel (Hyftor), using 20 gm per month. **C,** Sustained improvement after 9 months of treatment, using 10 gm per month. **D,** Residual hyperpigmentation consistent with erythromelanosis follicularis faciei et colli, noted 1 week off treatment, with summer sun exposure.

A 15-year-old generally healthy male presented for evaluation of a 2-year history of prominent facial erythema and rough skin. Distress about the appearance and texture prompted use of multiple topical products, including oxymetazoline and tretinoin, as well as pulsed dye laser treatments, all with limited efficacy. His skin exam featured symmetric patterned moderate erythema on both cheeks with associated densely scattered pinpoint keratotic papules characteristic of KPRF (Fig 1, *A*). He was prescribed twice daily topical treatment with sirolimus 0.2% gel (Hyftor). After using 20 gm/month for 3 months, he was very pleased with improvement in both appearance and flushing and denied adverse effects (Fig 1, *B*). The condition remained in remission for 6 more months, using only 10 gm/month (Fig 1, *C*). He was seen in follow up 1 month later after being off the medication for 1 week due to insurance denial for additional refills. This visit was in July, and only residual hyperpigmentation was noted, likely related to sun exposure and consistent with erythromelanosis follicularis faciei et colli (Fig 1, *D*).

## Discussion

Sirolimus was initially recognized for its antifungal properties; however, additional characterization identified potent antiproliferative and immunosuppressive effects via affinity for the cytoplasmic receptor FK506 binding protein-12, forming a complex that inhibits mammalian target of rapamycin expression, arresting the cell cycle.^4^^,^^6^ Mammalian target of rapamycin inhibition also downregulates smooth muscle and endothelial proliferation, reducing local inflammation and hyperkeratosis.^4^ In addition to treating angiofibromas and vascular malformations, oral sirolimus has been used off-label for a variety of other cutaneous conditions, including plexiform neurofibromas, scleromyxedema, and dermatomyositis.^4^^,^^5^

KPRF is often familial but typically an isolated cutaneous concern. An associated gene mutation has not been identified, but KPRF and a similar condition, ulerythema ophorygenes, have been associated with heritable diseases involving mutations in the rat sarcoma pathway/mitogen-activated protein kinase pathway, including Noonan, Cornelia de Lange, and cardiofacial cutaneous syndrome. Erythromelanosis follicularis faciei et colli is another keratosis pilaris variant that disproportionately affects skin-of-color.^7^

The appearance of KPRF can cause significant distress, impacting quality of life. A uniformly effective treatment has not been defined. Commonly recommended topical options of limited value include emollients, keratolytics, corticosteroids, and retinoids.^4^ While pulsed dye laser has been reported to reduce erythema, access to this treatment is limited and not often covered by insurance.^2^, ^3^, ^4^ This is the second reported case documenting excellent response to topical sirolimus, and the first using the commercially available 0.2% gel, supporting the value of a clinical trial.

## Conflicts of interest

Author Snow and Drs Ong and Siegfried are collaborating with NobelPharma on a funded related survey study to identify the unmet need of treating KPRF.
